# Genomic and pathological heterogeneity in clinically diagnosed small cell lung cancer in never/light smokers identifies therapeutically targetable alterations

**DOI:** 10.1002/1878-0261.12673

**Published:** 2020-11-25

**Authors:** Atsuko Ogino, Jihyun Choi, Mika Lin, Margaret K. Wilkens, Antonio Calles, Man Xu, Anika E. Adeni, Emily S. Chambers, Marzia Capelletti, Mohit Butaney, Nathanael S. Gray, Prafulla C. Gokhale, Sangeetha Palakurthi, Paul Kirschmeier, Geoffrey R. Oxnard, Lynette M. Sholl, Pasi A. Jänne

**Affiliations:** ^1^ Department of Medical Oncology Dana‐Farber Cancer Institute Boston MA USA; ^2^ Experimental Therapeutics Core Dana‐Farber Cancer Institute Boston MA USA; ^3^ Belfer Center for Applied Cancer Science Dana‐Farber Cancer Institute Boston MA USA; ^4^ Department of Biological Chemistry and Molecular Pharmacology Harvard Medical School Boston MA USA; ^5^ Department of Cancer Biology Dana‐Farber Cancer Institute Boston MA USA; ^6^ Lowe Center for Thoracic Oncology Dana‐Farber Cancer Institute Boston MA USA; ^7^ Department of Pathology Brigham and Women’s Hospital Boston MA USA

**Keywords:** combination therapy, lung cancers in never smokers, non‐neuroendocrine SCLC, *RAS* mutation, small‐cell lung cancer

## Abstract

Small‐cell lung cancer (SCLC) occurs infrequently in never/former light smokers. We sought to study this rare clinical subset through next‐generation sequencing (NGS) and by characterizing a representative patient‐derived model. We performed targeted NGS, as well as comprehensive pathological evaluation, in 11 never/former light smokers with clinically diagnosed SCLC. We established a patient‐derived model from one such patient (DFCI168) harboring an *NRAS* ^Q61K^ mutation and characterized the sensitivity of this model to MEK and TORC1/2 inhibitors. Despite the clinical diagnosis of SCLC, the majority (8/11) of cases were either of nonpulmonary origin or of mixed histology and included atypical carcinoid (*n* = 1), mixed non‐small‐cell lung carcinoma and SCLC (*n* = 4), unspecified poorly differentiated carcinoma (*n* = 1), or small‐cell carcinoma from different origins (*n* = 2). *RB1* and *TP53* mutations were found in four and five cases, respectively. Predicted driver mutations were detected in *EGFR* (*n* = 2), *NRAS* (*n* = 1), *KRAS* (*n* = 1), *BRCA1* (*n* = 1), and *ATM* (*n* = 1), and one case harbored a *TMPRSS2‐ERG* fusion. DFCI168 (*NRAS* ^Q61K^) exhibited marked sensitivity to MEK inhibitors *in vitro* and *in vivo.* The combination of MEK and mTORC1/2 inhibitors synergized to prevent compensatory mTOR activation, resulting in prolonged growth inhibition in this model and in three other *NRAS* mutant lung cancer cell lines. SCLC in never/former light smokers is rare and is potentially a distinct disease entity comprised of oncogenic driver mutation‐harboring carcinomas morphologically and/or clinically mimicking SCLC. Comprehensive pathologic review integrated with genomic profiling is critical in refining the diagnosis and in identifying potential therapeutic options.

## Graphical abstract

Small‐cell lung cancer occurs infrequently in never/former light smokers. We performed targeted NGS, as well as comprehensive pathological evaluation, in 11 never/former light smokers with clinically diagnosed SCLC. The majority of cases were either of nonpulmonary origin or of mixed histology. Predicted driver mutations were detected in *EGFR*, *NRAS*, *KRAS, BRCA1*, and *ATM,* and one case harbored a *TMPRSS2‐ERG* fusion.

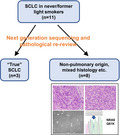

**Abbreviations**
EGFR: epidermal growth factor receptor
NE: neuroendocrine
NSCLC: non‐small‐cell lung cancer
PDX: patient‐derived xenografts
SCLC: small‐cell lung cancer

## 1 Introduction

Small‐cell lung cancer (SCLC) is one of the most challenging cancers to treat, with a 5‐year survival rate of 4–5% (Harris *et al.*, 2012). The standard therapy regimen for SCLC consists of platinum doublet chemotherapy, which has not changed over decades (Rudin *et al.*, 2019). SCLC is known to be strongly correlated with tobacco consumption; however, 2–3% of SCLC patients are reported to be never smokers (Ou *et al.*, 2009; Varghese *et al.*, 2014). Several studies have suggested that SCLC arising in never smokers includes patients with potentially actionable molecular aberrations (Sun *et al.*, 2015; Varghese *et al.*, 2014), yet optimal treatment modalities in this group have not been established due to the rarity of the cases, thus highlighting the urgent need to examine this possible biologically distinct subtype of SCLC to find novel therapeutic approaches.

Small‐cell lung cancer tumors consist of small, round‐shaped cells with a scant cytoplasm, fine granular nuclear chromatin, and frequent nuclear molding (Travis, 2012). The morphological characteristics of SCLC are distinct, and the diagnosis is mainly based on histological features. However, morphologic overlap with other entities, including large‐cell neuroendocrine (NE) carcinoma, basaloid squamous cell carcinoma, and ‘small round blue cell tumors’ including lymphoma, poorly differentiated melanoma, and sarcomas may contribute to diagnostic error and justifies the use of immunohistochemistry (IHC) to confirm the diagnosis. NE markers such as neural cell adhesion molecule (NCAM) (CD56), chromogranin A, synaptophysin, and INSM1 are characteristically expressed in SCLC but are not entirely specific. Recently, several researchers proposed that SCLC without classical NE markers expression can be defined by differential expression of YAP1 (McColl *et al.*, 2017) and POU2F3 (Huang *et al.*, 2018), emphasizing the biological heterogeneity of this morphologic entity and suggesting a need for more comprehensive tumor profiling to better understand this heterogeneous disease.

Concomitant inactivation of *RB1* and *TP53* is nearly universal in SCLC (George *et al.*, 2015). Known and suspected driver mutations have also been detected in several genes including in *PTEN, SLIT2, EPHA7, FGFR1, BRAF, KIT, PIK3CA,CREBBP, EP300,* and *MLL* (George *et al.*, 2015; Peifer *et al.*, 2012). However, the low frequency of clinically actionable driver mutations hinders the successful application of targeted therapies in SCLC. A difference in the mutational profile in SCLC according to the smoking status has also been reported (Cardona *et al.*, 2019; Sun *et al.*, 2015; Varghese *et al.*, 2014). One study by Cardona et al. demonstrated that *EGFR*, *MET,* and *SMAD4* are more frequently mutated in never smokers, while *RB1, CDKN2A,* and *CEBPA* are more frequent in smokers (Cardona *et al.*, 2019).

*NRAS,* together with *KRAS* and *HRAS*, belongs to the *RAS* oncogene family and encodes a highly conserved small GTPase which regulates cell growth, proliferation, and differentiation (Shimizu *et al.*, 1983). *NRAS* is mutated in roughly 1% of lung cancers (Ding *et al.*, 2008; Ohashi *et al.*, 2013). To date, no case of SCLC driven by an oncogenic *NRAS* mutant has been reported on, possibly due to the lack of routine genomic profiling of patients with SCLC. Currently, SW1271 is the only commercially available SCLC cell line with an *NRAS*‐activating mutation. SCLC harboring *HRAS* and *KRAS* mutations is also very uncommon (Rudin *et al.*, 2012; Varghese *et al.*, 2014). The rarity of these SCLC genotypes translates to a poor understanding of its biology and contributes to an ongoing debate on whether SCLC patients with RAF‐MEK‐ERK pathway activation can benefit from targeted therapies (Cristea and Sage, 2016).

In the current study, we evaluate a series of clinically diagnosed pulmonary SCLCs in never/light smokers and demonstrate the potential utility of combined MEK/mTORC1/2 inhibition in an exemplary case with *NRAS* ^Q61K^ mutation. We demonstrate that careful genetic, morphologic, and *in vitro* characterization highlights the diagnostic ambiguity of clinical SCLC and the essential role for tumor genomic profiling in SCLC arising in this rare clinical context.

## 2 Materials and Methods

### 2.1 Patients

Tumor biopsies from 19 treatment naïve SCLC patients who were either never smokers (*n* = 11) or light former smokers (≤ 10 pack‐years, *n* = 8) were analyzed for *NRAS* mutations alone by Sanger sequencing (*n* = 8) or comprehensively by targeted next‐generation sequencing (*n* = 11; OncoPanel (Garcia *et al.*, 2017)). All patients provided written informed consent for the analysis of their clinical specimens, and the studies were approved by the Institutional Review Board at Dana‐Farber Cancer Institute (DFCI). The study methodologies conformed to the standards set by the Declaration of Helsinki.

### 2.2 Cell cultures and Reagents

SW1271, H1299, H2087, H2347, H69, H82, H209, and Glc16 were purchased from American Type Culture Collection (ATCC). All cell lines except SW1271 were maintained in Roswell Park Memorial Institute (RPMI)‐1640 (Gibco, Thermo Fisher Scientific, Waltham, MA, USA) supplemented with 10% FBS, 100 U·mL^−1^ penicillin, and 100 µg·mL^−1^ streptomycin (Gibco, Thermo Fisher Scientific). SW1271 was grown in Dulbecco’s modified Eagle medium (Gibco, Thermo Fisher Scientific) with 10% FBS. Trametinib (Yamaguchi *et al.*, 2011), selumetinib (Davis *et al.*, 2011), INK128 (Hsieh *et al.*, 2012), AZD8055 (Chresta *et al.*, 2010), PI103 (Raynaud *et al.*, 2009), BKM120 (Burger *et al.*, 2011), ZSTK474 (Kong and Yamori, 2007), and MK2206 (Hirai *et al.*, 2010) were purchased from Selleck Chemicals (Houston, TX, USA). Torin2 was synthesized using previously published methods (Liu *et al.*, 2013). Stock solutions of all drugs were prepared at 10 mm in DMSO (Sigma‐Aldrich, St. Louis, MO, USA) and stored at −80 °C. Cell lines were authenticated by single tandem repeat analysis at Michigan State University in October 2017 and tested negative for mycoplasma as determined by the Mycoplasma Plus PCR Primer Set (Agilent Technologies, Santa Clara, CA, USA).

### 2.3 Generation of a patient‐derived cell line (DFCI168)

A pleural effusion was obtained from a patient with SCLC. The sample was subjected to red blood cell lysis using RBC Lysis Buffer (Boston BioProducts, Ashland, MA, USA), and the cells were suspended in HITES medium (Simms *et al.*, 1980) with 10% FBS. We carefully monitored the appearance of the cells during serial passage to minimize the loss of intratumor heterogeneity. Nevertheless, no floating cell aggregates which are the typical morphology of SCLC cell lines were detected throughout the course of cell line creation. Due to the unique cancer cell morphology resembling fibroblasts, the NCAM marker was used to positively select cancer cells using MACS columns (Miltenyi Biotechnology, Auburn, CA, USA). NCAM‐positive cancer cell population was subsequently cloned by limited serial dilution. The established cell line DFCI168 was maintained in RPMI‐1640 media supplemented with 10% FBS.

### 2.4 Cell proliferation and Growth assays, combination index

Inhibition of growth by targeted kinase inhibitors was evaluated by MTS assay according to the manufacturer’s instructions (Promega, Madison, WI, USA). Cells were plated in 96‐well plates at a density of 2000–8000 per well and treated on the following day. At 72 h after drug addition, cell viability was measured. Combination index (CI) was calculated using compusyn software (Combo Syn, Inc,. Paramus, NJ, USA). For *NRAS* siRNA knockdown experiments, cell viability was measured using CellTiter‐Glo luminescent assay (Promega).

### 2.5 Patient‐derived xenograft (PDX) establishment

To establish the DFCI168 PDX model, ~ 5 × 10^6^ cells from pleural effusion were implanted subcutaneously in 8‐week‐old female NSG mice in accordance with the guidelines approved by the DFCI Institutional Animal Care and Use Committee (IACUC) and tumor growth was monitored by caliper measurements. Once tumors grew to a size of 1 cm^3^, tumors were isolated and cut into pieces of ~ 2 × 2 × 2 mm and transplanted subcutaneously in additional NSG mice. Tumors were passaged for no more than five times. Samples from all passages were viably frozen in liquid nitrogen and used for further experiments. The tumor fidelity from various passages was confirmed by Hematoxylin and eosin (H&E) staining and the existence of the *NRAS* ^Q61K^ mutation.

### 2.6 Antitumor activity *in vivo*

All *in vivo* studies were conducted at DFCI with the approval of the Institutional Animal Care and Use Committee in an AAALAC‐accredited vivarium. The DFCI168 tumor fragments were transplanted subcutaneously on the right flanks of 8‐week‐old female NSG mice. Tumors were allowed to establish to 219 ± 54 mm^3^ in size before randomization into vehicle‐treated, trametinib‐treated (3 mg·kg^−1^, PO qd), torin2‐treated (30 mg·kg^−1^, PO qd), and combo‐treated groups (3 and 30 mg·kg^−1^, respectively) of 8 mice per group. Trametinib was administered in 0.5% hydroxypropyl methyl cellulose with 0.2% Tween 80. Torin2 was dissolved in captisol (1 : 40) followed by dilution in water to 3 mg·mL^−1^. Tumor volumes were determined from caliper measurements by using the formula V = (length × width^2^)/2. Tumor sizes and body weights were measured twice weekly. For pharmacodynamic (PD) study, mice were treated for 2 days and tumor samples were collected at 4 h (*n* = 3) after the last dose. Tumor samples were analyzed by western blot.

### 2.7 Flow cytometry experiments

NCAM/epithelial cell adhesion molecule (EpCAM) expression in the DFCI168 cells was evaluated with anti‐human NCAM APC antibody/anti‐human EpCAM‐PE antibody or with fluorophore‐tagged isotype control (eBioscience, Thermo Fisher Scientific). Early and late apoptotic cell death was assessed by Annexin V‐FITC/PI (Life Technologies, Thermo Fisher Scientific) or Annexin V‐PE/7AAD staining (BD Biosciences) according to the manufacturer’s protocol. The samples were analyzed with a BD LSR Fortessa flow cytometer with BD facsdiva software (BD Biosciences, Billerica, MA, USA).

### 2.8 Immunofluorescence staining

The DFCI168 cells were plated in 8‐well chamber slides at a concentration of 3 × 10^4^ cells per well. Two days after plating, cells were fixed with 4% paraformaldehyde for 10 min, followed by permeabilization with 0.5% Triton X‐100 for 10 min. After blocking with 10% goat serum for 1 h, the cells were stained with Phalloidin Green 488 (1 : 500; BioLegend, San Diego, CA, USA). For vimentin staining, the following antibodies and dilutions were used: rabbit anti‐vimentin (1 : 1000; Abcam, Cambridge, MA, USA) and goat anti‐rabbit Alexa Fluor 594 (1 : 1000; Invitrogen, Thermo Fisher Scientific). Slides were mounted with antifade mounting media containing DAPI. Images were obtained using the Nikon Eclipse 80i microscope (Nikon, Melville, NY, USA).

### 2.9 Sanger sequence and OncoPanel

The OncoPanel assay surveys exonic DNA sequences of 275 cancer genes and 91 introns across 30 genes for rearrangement detection. DNA was isolated from *NRAS* mutant cell lines using DNeasy Blood & Tissue Kit (Qiagen, Germantown, MD, USA) and analyzed by massively parallel sequencing using a solution‐phase Agilent SureSelect hybrid capture kit and an Illumina HiSeq 2500 sequencer. Further details were described elsewhere (Sholl *et al.*, 2016).

### 2.10 Plasmid construction and viral infection

pBabe *NRAS* ^Q61K^ plasmid was a gift from Channing Der (Addgene plasmid # 12543) (Khosravi‐Far *et al.*, 1996). pBabe *NRAS* ^Q61K^ plasmid or pBabe puro empty plasmid and a packaging plasmid pAmpho were co‐transfected into HEK293T cells. Viral supernatants were harvested at 48 h, filtered through 0.45‐µm filter, and spinoculation was performed by spinning at 1000 ***g*** for 90 min. After 48 h, cells were selected in puromycin (1 µg·mL^−1^).

### 2.11 Antibodies and immunoblotting

Cells were grown and treated as described and lysed with RIPA buffer with Triton X‐100 (Boston BioProducts) supplemented with protease and phosphatase inhibitors. Immunoblotting was performed according to the antibody manufacturers’ recommendations. The following antibodies were obtained from Cell Signaling Technology (Danvers, MA, USA): Anti‐Synaptophysin (SYP), phospho‐ERK1/2 (Thr202/Tyr204), ERK1/2, phospho‐RSK (Thr359/Ser363), RSK, phospho‐S6 (Ser235/236), phospho‐S6 (Ser240/244), S6, phospho‐4EBP (Thr37/46), 4EBP, phospho‐Akt (Ser473), Akt, Hsp90, phospho‐RB (Ser780), phospho‐RB (Ser807/811), RB, Vimentin, ZEB1, Snail, and Lamin B1. Anti‐ASCL1 antibody was purchased from BD Biosciences. Anti‐tubulin antibody is purchased from Sigma‐Aldrich. Anti‐NRAS antibody is purchased from Abcam.

### 2.12 siRNA experiments

Cells were transfected with 25 nm of control siRNA pool (D‐001206‐13‐05) or with *NRAS* specific siRNA pool (Dharmacon, Horizon Discovery, Lafayette, CO, USA). After 48 h, cell viability was measured by CellTiter‐Glo. *NRAS* silencing efficiency was verified by immunoblotting.

### 2.13 RNA isolation and quantitative real‐time PCR (qPCR)

RNA was extracted using TRIzol (Invitrogen) and column‐purified using RNeasy Kit (Qiagen). cDNA was generated from 1 µg of RNA using QuantiTect Reverse Transcription Kit (Qiagen). qPCR was performed and analyzed on Step One Plus Real‐time PCR System using TaqMan probes (Applied Biosystems, Thermo Fisher Scientific) for E‐cadherin (Hs01023894), vimentin (Hs00958111), SNAI1 (Hs00195591), TWIST (Hs00361186), ZEB1 (Hs00232783), ASCL1 (Hs00269932), and SYP (Hs00300531). GUSB was used as a housekeeping gene.

## 3 Results

### 3.1 Pathologic reassessment and genomic testing of clinically diagnosed SCLC patients who are never and light former smokers (≤ 10 pack‐years)

We identified 11 cases of never (*n* = 7) or light former (*n* = 4) smokers with clinically diagnosed SCLC treated at DFCI from 2013 to 2018 (Table 1). Mutational analysis was performed using the targeted next‐generation sequencing (NGS) profiling platform, OncoPanel (Garcia *et al.*, 2017) (Tables 1 and S1). Four cases harbored mutations in both *RB1* and *TP53*, and one had a mutation only in *TP53*. Among *RB1* wild‐type (WT) cases (*n* = 7), two cases were positive for *EGFR* mutations (case 8: L858R and L861F, case 10: L858R), and the others harbored *NRAS* ^Q61K^ (*n* = 1), *BRCA1* ^L502Afs*2^ (*n* = 1), and *ATM* ^T1953I^ (*n* = 1) and high *MET* amplification (*n* = 1). In *RB1* mutant cases (*n* = 4), *KRAS* ^G12V^ (*n* = 1) and *TMPRSS2‐ERG* fusion (*n* = 1) were detected. An additional eight patients with SCLC (never smokers (*n* = 4) or light former smokers (*n* = 4)) with only limited DNA were specifically queried for an *NRAS* mutation, but no *NRAS* mutations were detected (data not shown).

**Table 1:** Clinicopathologic characteristics of 11 clinically diagnosed never/light smokers with SCLC. LCNEC, large‐cell neuroendocrine cancer; mt, mutant; ND, not done.

Patient number	Age	Sex	Smoking (pack‐years)	Pathology diagnosis	RB1/TP53 status	RB protein expression	Myc status
1	68	F	0	Combined LCNEC and SCLC	RB1 WT/TP53 WT	ND	
2	50	F	0	Combined LCNEC and SCLC	RB1 WT/TP53 WT	Intact	
3	60	M	0	Neuroendocrine tumor grade 2/atypical carcinoid, pancreas, or lung primary	RB1 WT/TP53 WT	Intact	
4	52	F	0	Small cell of unknown primary hepatobiliary origin suspected	RB1 mt/TP53 mt	ND	Low copy number gain
5	70	M	0	SCLC	RB1 mt/TP53 mt	ND	Low copy number gain
6	59	F	0	SCLC	RB1 WT/TP53 WT	ND	
7	50	M	0	Poorly differentiated carcinoma	RB1 WT/TP53 WT	Intact	
8	47	F	2	NSCLC undergoing *de novo* SCLC transformation	RB1 WT/TP53 WT	ND	
9	51	F	4	SCLC	RB1 mt/TP53 mt	ND	Myc, MycL gain
10	62	F	7	NSCLC undergoing *de novo* SCLC transformation	RB1 WT/TP53 mt	ND	
11	76	M	10	Metastatic prostate cancer, with small‐cell transformation	RB1 mt/TP53 mt	ND	Myc gain

Due to the identification of mutations infrequently found in typical SCLC patients, microscopic features were retrospectively re‐evaluated. The review of two cases harboring *EGFR* mutations (case 8 and case 10) showed admixed non‐small‐cell and small‐cell features, suggestive of *de novo* small‐cell transformation of *EGFR*‐mutated adenocarcinomas. Case 1 with an *ATM* mutation and case 2 with a *MET* amplification showed features intermediate between large‐cell NE and small‐cell carcinomas. Case 7 with a *NRAS* mutation was verified to be a keratin‐positive small round blue cell tumor but lacked any specific features to confirm a diagnosis of SCLC. A *BRCA1* mutation‐harboring case 3 was reclassified as grade 2 NE tumor/atypical carcinoid of possible pancreas or lung primary in the available clinical and radiographic context. Among the five cases with classic small‐cell carcinoma histology, case 4 with *KRAS* ^G12V^ was suspected to be of possibly of hepatobiliary origin. Case 11 harbored a *TMPRSS2‐ERG* fusion, an oncogenic fusion common in prostate cancers (Attard *et al.*, 2008; Demichelis *et al.*, 2007) without any reported occurrences in lung cancer. Given the patient’s prior history of prostate carcinoma, this case was reclassified as prostate cancer with small‐cell transformation. Thus, following pathological re‐review, 3/11 (27%) cases remained as SCLC. Of these, two cases had mutations in both *TP53* and *RB1* (Table 1).

### 3.2 Identification of NRAS^Q61K^ mutation in clinically diagnosed SCLC and establishment of patient‐derived cancer models

**Fig. 1:**
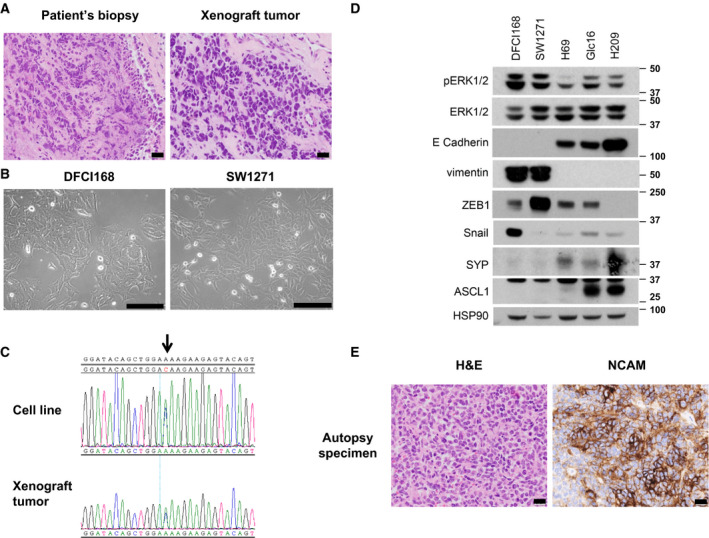
DFCI168 harbors unique non‐NE features. (A) H&E staining (20×) of patient tumor (left) and mouse xenograft tumor derived from pleural effusion from the patient (right). Scale bar, 20 µm (B) Phase‐contrast images of DFCI168 and SW1271. Scale bar, 100 µm (C) Nucleotide sequence tracings showing *NRAS* ^Q61K^ mutation (indicated by arrow) in both the DFCI168 cell line and the xenograft tumor. (D) Western blot analysis of phospho‐ERK1/2, ERK1/2, Vimentin, and NE markers (ASCL1 and SYP) and Hsp90 (loading control). (E) H&E staining (left, 20×) and IHC for NCAM (right, 20×) of autopsy specimens from the patient. Scale bar, 20 µm.

We next developed both *in vitro* and *in vivo* models from patient 7 (Table 1) harboring a *NRAS* somatic mutation at codon 61 (Q61K). The patient is a 49‐year‐old male who presented with a large right hilar mass (Fig. S1). Although he had no history of cigarette smoking, he had a history of second‐hand smoking from both parents who were heavy smokers. The histology of the endobronchial biopsy demonstrated morphological features of SCLC, presenting small‐sized cells with a high nuclear‐to‐cytoplasmic ratio, nuclear molding, and frequent single‐cell apoptosis (CK7+, NCAM+, SYP−, Chromogranin−) (Fig. 1A, left, and data not shown). The lack of the expression of some common NE markers (SYP, Chromogranin) together with the unusual genomic features suggests that the tumors of this patient do not possess the features of the classic type of SCLC.

With a clinical diagnosis of limited‐stage disease SCLC, this patient was treated with concurrent chemoradiation therapy, which led to a complete response. Despite the good response, he developed widespread metastatic disease just several months after chemoradiotherapy and could not receive any further systemic treatment due to a declining clinical condition.

A week prior to the patient’s passing, pleural effusion was obtained and established into a cell line, DFCI168. To investigate the characteristics of *NRAS* mutant SCLC, a PDX model was also established by subcutaneous implantation of cancer cells from the same pleural effusion. The tumor xenografts retained morphological features of SCLC (Fig. 1A, right). The xenograft mice developed spontaneous lung metastases, mimicking the aggressive biology of the patient’s tumor.

Unlike most SCLC cell lines that grow in culture as floating aggregates, DFCI168 grows in a monolayer and displays a spindle‐shaped morphology (Fig. 1B, left). We next assessed SW1271, a commercially available SCLC cell line with a *NRAS* ^Q61R^ mutation. The morphological features of SW1271 were similar and consistent with DFCI168 (Fig. 1B, right). *TP53* was mutated (p. C277F), but no *RB1* mutation was detected by OncoPanel in SW1271. The *NRAS* ^Q61K^ mutation was confirmed in both the xenograft tumor and the DFCI168 cell line by Sanger sequencing (Fig. 1C). Flow cytometric analysis revealed that DFCI168 was EpCAM‐negative and NCAM‐positive (Fig. S2A). Consistent with the absence of mutations in *RB1*, RB1 protein expression was maintained in DFCI168 and SW1271 (Fig. S2B). DFCI168 and SW1271 express vimentin and either ZEB1 or Snail, transcription factors known to regulate EMT with concomitant downregulation of E‐cadherin, while the expression of NE markers (SYP and ASCL1) is almost undetectable (Figs 1D and S2D). Due to the genotypic and phenotypic discrepancy between DFCI168 and classic SCLC, we carefully examined the histology of autopsy‐retrieved specimens from this patient. Although the tissue demonstrated patchy‐positive NCAM staining (Fig. 1E, right), the autopsy material showed increased cytoplasm as compared to the original biopsy, striking nuclear pleomorphism and distinct nucleoli (Fig. 1E, left), which do not represent the typical histological characteristics of SCLC. Architectural features of large‐cell NE carcinoma were also lacking. We further investigated the possibility of an alternative diagnosis including either a rhabdomyosarcoma or a melanoma, both of which have recurrent *NRAS* mutations, but the tissue was negative for all markers specific to these tumor types (SOX10, S100, myf4, and desmin). At autopsy, this tumor was best classified as an undifferentiated carcinoma with NE differentiation by IHC, presenting clinically and being treated as SCLC.

### 3.3 DFCI168 cells depend on NRAS signaling for survival and show high sensitivity to MEK inhibition

**Fig. 2:**
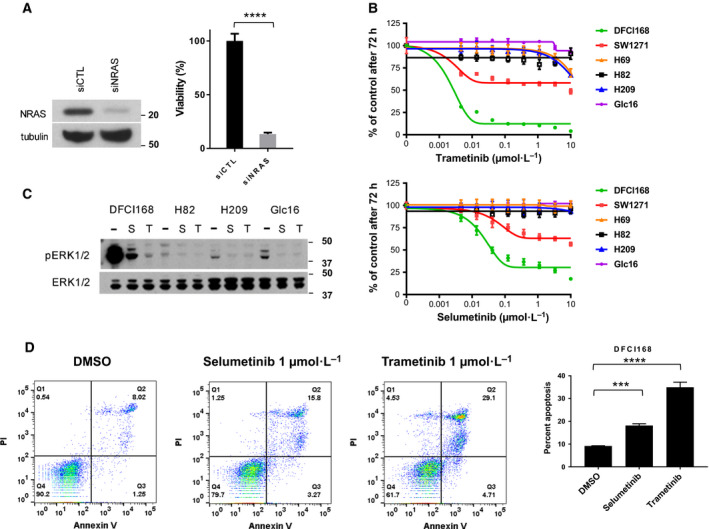
DFCI168 depends on NRAS signaling for survival. (A) DFCI168 cells were transfected with siRNA pool directed against *NRAS* or with control siRNA pool. The efficiency of NRAS knockdown was confirmed by western blot after 48 h post‐transfection. The cell viability was assessed by CellTiter‐Glo (*n* = 5, error bars represent the SD of the mean) at 48 h post‐transfection. Significance was assessed by unpaired *t*‐test. ****P < 0.0001. (B) The SCLC cell lines were treated with increasing doses of MEK inhibitors (trametinib or selumetinib), and the cell viability was assessed by MTS after 72 h (*n* = 6, mean ± SD). (C) The SCLC cell lines were treated with 1 µm selumetinib (S) or trametinib (T) 100 nm for 6 h, and then, ERK1/2 phosphorylation was evaluated by western blotting. (D) DFCI168 was treated with 1 µm selumetinib or 1 µm trametinib for 48 h. The cells were stained with Annexin V‐PE/7‐AAD (7‐aminoactinomycin D) and analyzed by flow cytometry. The lower right quadrant shows early apoptotic cells, and the upper right quadrant represents late apoptotic cells. The numbers in the graph represent the percentage of cell numbers in each quadrant. Bar graphs represent the mean of percentage from three independent experiments. Significance was assessed by one‐way ANOVA. ***P < 0.001, ****P < 0.0001.

Next, we sought to determine whether the DFCI168 cells depend on the NRAS pathway for their survival using siRNA‐mediated *NRAS* knockdown (Fig. 2A, left). NRAS downregulation resulted in a significant decrease in cell viability of the DFCI168 cells (*P* < 0.0001, Fig. 2A, right). We further assessed the sensitivity of DFCI168 and SW1271 to MEK inhibitors and compared them to *NRAS* WT SCLC cell lines (H82, H209, Glc16). Both trametinib and selumetinib showed selective potency in *NRAS* mutant cell lines (IC_50_ in DFCI168: trametinib 2 nm; selumetinib 50 nm), but not in *NRAS* WT SCLC cell lines (Fig. 2B), despite ERK phosphorylation being significantly suppressed upon MEK inhibition in both SCLC subtypes (Fig. 2C). To verify the apoptotic effects of MEK inhibition on DFCI168 cells, we performed flow cytometry analysis of Annexin V and 7AAD staining. Treatment with 1 µm of selumetinib or trametinib for 48 h significantly increased apoptotic cell population as compared to DMSO. (*P* = 0.001; one‐way ANOVA) (Fig. 2D).

### 3.4 The diversity in sensitivity to MEK inhibition in NRAS mutant lung cancer cell lines

**Fig. 3:**
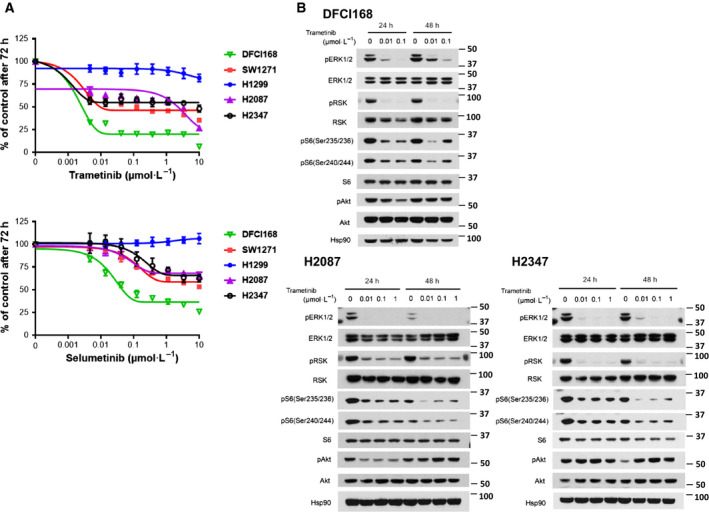
Heterogeneity in response to MEK inhibitors in *NRAS* mutant lung cancer cell lines. (A) The *NRAS* mutant lung cancer cell lines were treated with increasing doses of MEK inhibitors (trametinib and selumetinib). The cell viability was assessed by MTS after 72 h. (B) DFCI168, H2087, and H2347 were treated with trametinib at the indicated concentration for the indicated times. The cell extracts were immunoblotted using the indicated antibodies.

To further investigate the dependence of lung cancer harboring the *NRAS* ^Q61^ mutation on MEK signaling, we tested three *NRAS* mutant non‐small‐cell lung cancer (NSCLC) lines (H1299:*NRAS* ^Q61K^, large‐cell carcinoma; H2087:*NRAS* ^Q61K^, adenocarcinoma; H2347:*NRAS* ^Q61R^, adenocarcinoma) for sensitivity to MEK inhibitors. H1299 showed resistance (IC_50_ > 10 µm) to trametinib and selumetinib in the cell viability assay (Fig. 3A), which may be attributed to its dependence on PI3K/Akt signaling for survival due to a PTEN promoter methylation (Soria *et al.*, 2002).

H2087 and H2347 showed limited sensitivity to MEK inhibition similar to SW1271, with > 30% of the cells still viable at 10 µm of MEK inhibitor treatment. To elucidate the mechanism underlying the difference in sensitivity to MEK inhibition between the *NRAS* mutant cell lines, we investigated the downstream effectors of RAS, Akt/mTOR, and ERK. The basal level of p‐ERK varied across the tested *NRAS* mutant cell lines, exhibiting no obvious trend to predict MEK inhibitor sensitivity. Although high p‐Akt expression is a reported predictor of resistance to MEK inhibition in melanoma patients (Atefi *et al.*, 2011; Catalanotti *et al.*, 2013; Gopal *et al.*, 2010), the p‐Akt level was highest in DFCI168, suggesting that sensitivity to MEK inhibitor treatment does not correlate with the level of p‐Akt in the cell lines we tested (Fig. S2C).

### 3.5 The combination of MEK and mTORC1/2 inhibitors synergizes to sustain growth inhibition of NRAS mutant lung cancer cells

In order to identify signaling pathways that may compensate for MEK inhibition, and as such explain the diversity of responses to single‐agent MEK inhibition, we analyzed PI3K/Akt and mTOR signaling in the *NRAS* mutant NSCLC cell lines in the presence of a MEK inhibitor. S6 phosphorylation, especially at residues 240/244, serves as a marker of mTORC1 signaling (Elkabets *et al.*, 2013; Roux *et al.*, 2007). In all five cell lines, Akt and S6 activation persisted during MEK inhibitor treatment (Figs 3B and S3). In DFCI168 and H1299, the levels of phosphorylated S6 increased with higher concentrations of trametinib (100 vs. 10 nm in DFCI168, 1 µm vs. 10 and 100 nm in H1299) (Figs 3B and S3), suggesting feedback regulation between MEK/ERK and mTOR pathways without affecting the p‐Akt levels. These results prompted us to explore whether inhibition of PI3K/Akt/mTOR signaling can enhance cytotoxicity caused by MEK inhibition. To test this, we evaluated the effects of the dual PI3K/mTOR inhibitor (PI103), pan‐class I PI3K inhibitors (BKM120, ZSTK474), Akt inhibitor (MK2206), and mTORC1/2 inhibitors (INK128, torin2, AZD8055) as single agents. Among those tested, mTORC1/2 inhibitors were the most potent against *NRAS* mutant cell lines (Fig. S4). Importantly, all the *NRAS* mutant cell lines tested showed substantial sensitivity to single‐agent mTORC1/2 inhibitors (IC_50_ < 1 µm). We also confirmed that the combination of 100 nm of trametinib and torin2 abrogates the phosphorylation of S6 in DFCI168 and H2087 (Fig. S5).

**Fig. 4:**
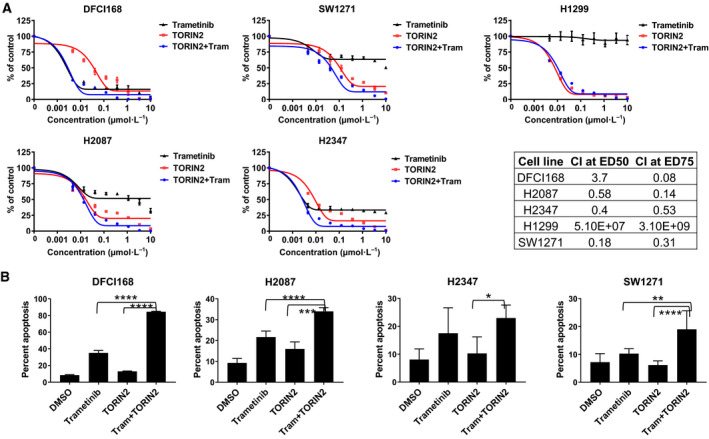
Evaluation of the combination of MEK and mTORC1/2 inhibitors on cell viability in *NRAS* mutant lung cancer cell lines. (A) The cell lines were treated with increasing doses of combination of trametinib and torin2 at the fixed ratio of 1 : 1. The cell viability was assessed by MTS after 72 h. The CI was calculated for 1 : 1 ratio of combined trametinib and torin2 and presented in the table. The CI values define synergism (CI < 1), additive effect (CI = 1), and antagonism (CI > 1), respectively. (B) The cell lines were treated for 48 h with 1 µm trametinib, 1 µm torin2, or the combination except for DFCI168 which was treated with 50 nm trametinib, 100 nm torin2, or the combination. The treated cells were stained either with Annexin V‐FITC/PI or with Annexin V‐PE/7AAD and analyzed by flow cytometry. Bar graphs represent the mean of percentage from three independent experiments. Significance was assessed by one‐way ANOVA. *P < 0.05, **P < 0.01, ***P < 0.001, ****P < 0.0001.

We further investigated the synergistic effects of the combination of MEK and mTORC1/2 inhibitors. MTS‐based cell viability assays were performed after a 72‐h treatment with trametinib and torin2, either alone or in combination at a constant ratio of 1 : 1 (Fig. 4A). No synergistic or additive effects of the combination treatment were observed in H1299, suggesting that this cell line depends solely on the PI3K/Akt/mTOR pathway for survival due to the known PTEN promoter methylation (Soria *et al.*, 2002). Unexpectedly, single‐agent torin2 showed greater potency than trametinib alone in four out of five cell lines (Fig. 4A). We also performed CI analysis according to the Chou–Talalay equation to determine the synergistic relationship between the two drugs (Fig. 4B). The CI values represent synergistic effects with CI < 1, additive effects with CI = 1, and antagonistic effect with CI > 1. Treated with combinations at a fixed ratio of 1 : 1, CI values were < 1 for H2087, H2347, and SW127 both at ED50 and ED75, and for DFCI168 at ED75. No synergistic effect of the combination was observed in H1299 (Fig. 4B).

To test whether the drugs induce apoptosis, the cell lines were treated with trametinib and torin2 for 48 h and stained with either FITC‐labeled Annexin V and PI‐ or PE‐labeled Annexin V and 7AAD (Fig. 4C). The combination significantly increased the apoptotic cell death compared with the single agents.

### 3.6 Combined treatment with MEK and mTORC1/2 inhibitors is effective in DFCI168 *in vitro* and *in vivo*

**Fig. 5:**
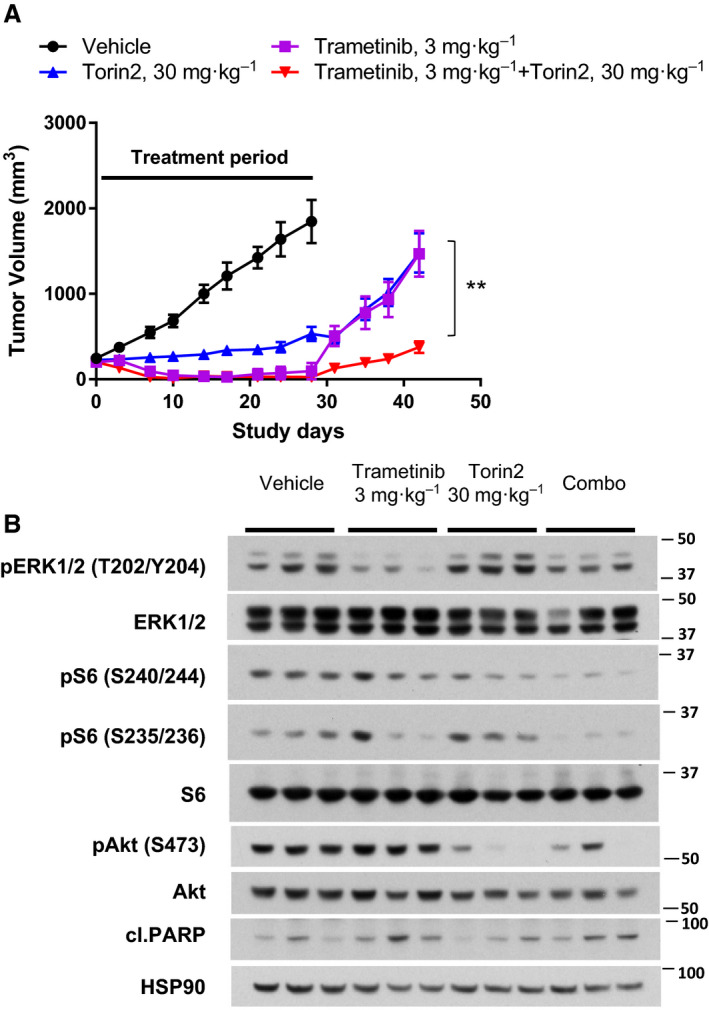
Combined treatment with trametinib and torin2 is effective in DFCI168 PDX tumors. (A) Tumor growth curves treated with vehicle (*n* = 8), trametinib (3 mg·kg^−1^, *n* = 8), torin2 (30 mg·kg^−1^, *n* = 8), and combination thereof (*n* = 7) with indicated 28 days of treatment period. Tumor volumes were recorded biweekly. Significance was assessed by one‐way ANOVA. **P < 0.01 comparing between single‐agent treatment and combination treatment. (B) The PD effect of trametinib and torin2. Mice were treated as indicated for 2 days, and tumor samples were collected at 4 h (*n* = 3) after the last dose.

Given the good response to single‐agent MEK inhibitors in DFCI168 *in vitro*, we further evaluated the effect of trametinib *in vivo* using a PDX model from DFCI 168. The DFCI168 tumor‐bearing mice were treated with trametinib at 3 mg·kg^−1^ daily for 3 weeks. The tumor initially regressed significantly but grew back within 4 weeks after treatment was stopped, likely from the residual cells not eliminated by drug treatment (Fig. 5A). Next, we tested the *in vivo* efficacy of the combination of trametinib and torin2 in the DFCI168 PDX model. The mice were treated with trametinib, torin2, or the combination for 28 days (Fig. 5A). Even though all treatments lead to significant tumor regression, all tumors eventually regrew after treatment cessation. The tumor regrowth following the combination treatment withdrawal was significantly slower than the following single‐agent treatment withdrawal (*P* < 0.01). While trametinib at 3 mg·kg^−1^ was well‐tolerated, torin2 at 30 mg·kg^−1^ treatment required a drug holiday in three out of eight mice. The combination treatment resulted in > 15% body weight loss in four out of seven mice, also requiring drug holidays. To confirm on‐target inhibition of the combination treatment in this model, we conducted a 2‐day PD study (Fig. 5B). The combination of trametinib and torin2 significantly suppressed the downstream targets of both MEK and mTOR pathways, p‐ERK1/2 (T202/Y204) and p‐S6 (both S240/244 and S235/235), respectively.

### 3.7 Ectopic expression of NRAS^Q61K^ induces non‐NE phenotype

**Fig. 6:**
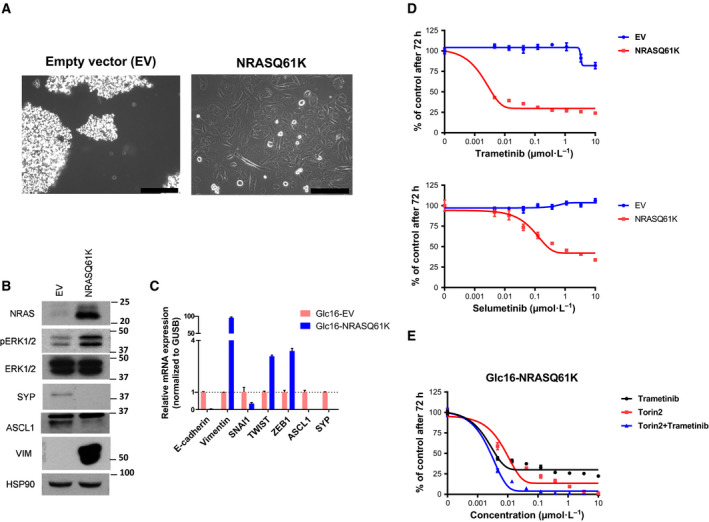
Transition from NE to non‐NE mediated by *NRAS* ^Q61K^. (A) Phase‐contrast images of Glc16 cells transduced with retrovirus containing *NRAS* ^Q61K^ or the empty vector. Scale bar, 100 µm. (B) The cell extracts were immunoblotted for the detection of indicated protein including NE markers (ASCL1 and SYP). (C) RNA was collected as well, and quantitative PCR was carried out using probes for indicated genes including mesenchymal markers and transcription factors (*E‐cadherin, vimentin, SNAI1, TWIST,* and *ZEB1*). (D) The empty vector/*NRAS* ^Q61K^‐transduced Glc16 cell lines were treated with increasing doses of trametinib or selumetinib, and the cell viability was assessed by MTS after 72 h (*n* = 6, mean ± SD). (E) The *NRAS* ^Q61K^ transduced Glc16 cell line was treated with increasing doses of trametinib or torin2 alone, or in combination, and the cell viability was assessed by MTS after 72 h (*n* = 6, mean ± SD).

Given the similarity in non‐NE mesenchymal features between DFCI168 and SW1271, we hypothesized that the *NRAS*‐activating mutation might play a role in the acquisition of this unique phenotype. To address this hypothesis, we retrovirally transduced the classical SCLC cell line, Glc16, with *NRAS* ^Q61K^. The *NRAS* ^Q61K^‐expressing Glc16 derivative became adherent, outstretched, and acquired a large elongated morphology similar to that of DFCI168 and SW1271 (Fig. 6A). *NRAS* mutant protein overexpression and ERK1/2 activation were confirmed by western blotting (Fig. 6B). Quantitative PCR analysis revealed an increase in mRNA expression of *vimentin*, *TWIST*, and *ZEB1*, and a decrease in expression of *E‐cadherin* in *NRAS* ^Q61K^ transduced cells, consistent with these cells having undergone EMT (Fig. 6C). Furthermore, the NE markers, ASCL1 and SYP, were also shown to have decreased in expression in these cells. Importantly, the *NRAS* ^Q61K^‐transduced cells acquired sensitivity to MEK inhibitors (Fig. 6D) and showed substantial response to the trametinib/torin2 combination (Fig. 6E).

## 4 Discussion

Small‐cell lung cancer is an aggressive malignancy most often presenting in patients who are former or current smokers. However, SCLC can occasionally be diagnosed in individuals who are never or light former smokers (Ou *et al.*, 2009; Varghese *et al.*, 2014). Diagnostic specimens in SCLC are generally small hence limiting comprehensive pathological and/or molecular analyses. Furthermore, given no clear targetable alterations in SCLC, these tumors often do not undergo extensive genomic analyses. Recent studies suggest that SCLC can be heterogeneous, consisting of both NE and non‐NE cells, which may warrant an alternative treatment approach (Gazdar *et al.*, 2017; Mollaoglu *et al.*, 2017; Rudin *et al.*, 2019). By both pathological re‐examination and molecular analyses of 11 cases of clinically diagnosed pulmonary SCLC in never/light smokers, our study highlights the biological heterogeneity within this group and that in a subset a nonpulmonary origin should also be considered despite the clinical diagnosis of lung origin. Intriguingly, even after pathological re‐review, not all of the SCLC tumors harbored *TP53* and *RB1* mutations, suggesting that SCLC in never/light smokers may also be distinct at the genetic level. However, additional studies are needed to support this hypothesis. Our study exemplifies the genomic and phenotypic heterogeneity of SCLC in never smokers and suggests that tumors which clinically and pathologically mimic SCLC can have strikingly different genomic underpinnings and phenotypic plasticity.

We successfully established a cell line and PDX (DFCI168) from a clinically diagnosed SCLC never smoker with a *NRAS* ^Q61K^ mutation (case 7; Table 1). Interestingly, DFCI168 and SW1271 both harbor *NRAS*‐activating mutations and exhibit characteristics distinct from classic SCLC, such as upregulation of mesenchymal markers and a lack of NE marker expression. Although *RB1* inactivation is highly characteristic of SCLC (> 90%) (George *et al.*, 2015), targeted next‐generation sequencing (OncoPanel) analyses revealed no *RB1* mutation in either DFCI168 or SW1271. DFCI168 was diagnosed and treated as a SCLC, although detailed molecular and histological analyses, including examination of postmortem specimens, could not definitively classify this as SCLC. SW1271 is listed as a SCLC cell line at the ATCC site (https://www.atcc.org/), but the similarities between SW1271 and DFCI168 raise a question whether SW1271 is in fact a true SCLC cell line. To date, SW1271 has been commonly used as a SCLC cell line in several prior studies (Dabir *et al.*, 2014; Greenberg *et al.*, 2015; Polley *et al.*, 2016) .

This rare subset of pulmonary SCLC in never/light smokers is of particular interest as druggable oncogenic alterations in NSCLC, including *EGFR* mutations and *ALK* rearrangements, are more commonly observed in never smokers than in those with a history of smoking (Shigematsu *et al.*, 2005; Wong *et al.*, 2009). We observed two cases of epidermal growth factor receptor (EGFR) inhibitor naïve *EGFR* mutant SCLC arising in the background of NSCLC (Table 1). Both patients were treated with erlotinib: One had no response, while the other had a sustained clinical response (data not shown). Two independent studies assessed SCLC samples from never smokers by NGS also identified *EGFR* mutations (6/36 cases), but detected no *RAS* mutations (Sun *et al.*, 2015; Varghese *et al.*, 2014). We further reviewed data from publicly available SCLC datasets using the cBioPortal website (http://www.cbioportal.org/) and found no *NRAS* mutations reported in the 210 SCLC cases regardless of smoking status. Considering that *NRAS* mutations in NSCLC are more frequent in previous/current smokers (Ohashi *et al.*, 2013), comprehensive genomic profiling of SCLC regardless of smoking status will be necessary to determine the true incidence of *NRAS* mutant SCLC.

We observed heterogeneity in response to MEK inhibitors in five *NRAS* mutant lung cancer cell lines. Among those tested, DFCI168 was the most sensitive to MEK inhibition. DFCI168 exhibits prominent EMT features, and its high sensitivity to MEK inhibition does not correlate with previous findings where EMT is often associated with drug resistance (Kitai *et al.*, 2016). Several studies using human and mouse cell lines have reported that RAS activation (H‐RAS and K‐RAS) could induce a non‐NE EMT phenotype (Calbo *et al.*, 2011; Falco *et al.*, 1990; Mabry *et al.*, 1988). Here, we show that ectopically expressed *NRAS* ^Q61K^, too, is sufficient to induce the acquisition of non‐NE characteristics in the classic SCLC cell line, Glc16. Furthermore, the Glc16 *NRAS^Q61K^* cells acquire sensitivity to MEK inhibition, analogous to DFCI168. However, not all of the *NRAS* mutant cell lines were sensitive to MEK inhibition. A recent study by Corcoran *et al.* (2013). revealed the importance of p‐S6, one of the markers of mTORC1 signaling, as a predictor of response to RAF and MEK inhibitors in BRAF mutant melanoma. We previously reported that the mTOR pathway not only serves as a predictive marker for sensitivity to the combination of the EGFR‐TKI inhibitor WZ4002 and trametinib, but also is involved in acquired resistance to this combination treatment in *EGFR* mutant lung adenocarcinoma (Tricker *et al.*, 2015). Trametinib treatment reduced the p‐S6 levels in the *NRAS* mutant cell lines we examined, but did not eliminate the activation of S6. In addition, in four out of five cell lines tested, mTORC1/2 inhibitors showed more potency than MEK inhibitors, further emphasizing the importance of inhibiting mTOR signaling in *NRAS* mutant cell lines. This finding is in agreement with a recent publication by Kiessling et al, who showed that the mTORC1/2 inhibitor, everolimus, alone was sufficient to inhibit cell growth in *NRAS* mutant neuroblastoma cell lines (Kiessling *et al.*, 2016). In our study, MEK and mTORC1/2 inhibitors exhibited a synergistic effect in 4 out of 5 *NRAS* mutant lung cancer cell lines. We also demonstrate that the trametinib and torin2 combination is significantly better at delaying the tumor regrowth *in vivo* than single agent alone, which is consistent with previous studies (Bailey *et al.*, 2014; Kiessling *et al.*, 2015; Vujic *et al.*, 2014). Many signaling pathways converge at mTOR, and the long‐term efficacy of the dual MEK/mTOR inhibition highlights mTOR as an attractive target for combination therapy. While tumor regrowth was slower after the withdrawal of the combination *in vivo*, it did not completely prevent tumor regrowth. This is possibly due to insufficient drug exposure, as the combination treatment did not completely inhibit p‐ERK1/2 and p‐S6 levels (Fig. 5). Additional studies, including intermittent dosing strategies, are needed to effectively combine these two agents to maximize pathway inhibition.

## 5 Conclusions

In summary, our findings highlight unique features of SCLC in never/light smokers. Given the aggressive clinical nature of SCLC and the often scant specimens available for pathological and molecular analyses, treatment is commonly based solely on H&E staining with the assistance of IHC and on clinical presentation. However, detailed pathological and molecular analyses reveal that clinically diagnosed SCLC in never/light smokers is in fact more heterogeneous with some cases harboring targetable genomic alterations. The identification and study of such cases may reveal new therapeutic options that are not typically used in classic SCLC. With the increased systematic use of comprehensive genomic sequencing, including in SCLC, more cases of this SCLC subtype may be identified, further underscoring the importance of establishing personalized treatment approaches for these patients.

## Conflict of interest

PAJ has received consulting fees from AstraZeneca, Boehringer Ingelheim, Pfizer, Roche/Genentech, Merrimack Pharmaceuticals, Chugai Pharmaceuticals, Takeda Oncology, Eli Lilly and Company, Araxes Pharma, Ignyta, Mirati Therapeutics, Novartis, LOXO Oncology, Daiichi‐Sankyo, Voronoi, SFJ Pharmaceuticals, and Biocartis; receives postmarketing royalties from DFCI owned intellectual property on EGFR mutations licensed to Lab Corp; has sponsored research agreements with AstraZeneca, Daiichi‐Sankyo, PUMA, Boehringer Ingelheim, Eli Lilly and Company, Takeda Oncology, and Astellas Pharmaceuticals; and has stock ownership in LOXO Oncology and Gatekeeper Pharmaceuticals. GRO has received consulting fees from AstraZeneca and Inivata and honoraria from Guardant. LMS has received consulting fees from Foghorn Therapeutics and LOXO Oncology and honoraria from AstraZeneca. AC has received honorary/consulting fees from AstraZeneca, Boehringer Ingelheim, Pfizer, Roche/Genentech, Eli Lilly and Company, Novartis, Merck Sharp & Dohme, and Bristol‐Myers Squibb.

All remaining authors have no conflicts of interest.

## Author contributions

AO and PAJ conceived and designed the project. AO, JC, MKW, AC, MX, and MC carried out the experiments, and analyzed and interpreted the data. NSG provided the study materials. AO, LMS, and PAJ wrote the paper. ML edited the manuscript. AEA, ESC, MB, and GRO provided the patient data/samples. PCG, SP, PK, and LMS contributed to the study design and data interpretation. PAJ supervised the findings of this work. All authors have read and approved the final version of the manuscript.

## Supporting information


**Fig. S1.** Treatment timeline: Dynamic contrast‐enhanced CT scan and FDG‐PET image are shown.

**Fig. S2.** Characterization of *NRAS* mutant lung cancer cell lines. (A) Flow cytometric analysis of NCAM/EpCAM expression on DFCI168. (B) (left) The lysates from nuclear fractions of lung cancer cell lines were examined for RB, pRB (Ser807/811) and lamin B (loading control). (right) IHC image of DFCI168 PDX stained with RB. Scale bar 50 µm. (C) The activation of PI3K/Akt/mTOR and MEK/ERK pathways in the *NRAS* mutant lung cancer cell lines was assessed by western blot. (D) Immunofluorescence images of DFCI168 stained with phalloidin (green) and VIM (red). Scale bars 100 µm.

**Fig. S3.** H1299 and SW1271 were treated with trametinib at the indicated concentration for the indicated times. The cell extracts were immunoblotted using the indicated antibodies.

**Fig. S4**. The comparison of IC50 values of various inhibitors for *NRAS* mutant lung cancer cell lines after 72 h of treatment. The results were obtained from three independent experiments, and the bar represents the mean ± SD.

**Fig. S5.** DFCI168 and H2087 were treated with 100 nm of trametinib/torin2 either alone or in combination for the indicated times. The cell extracts were immunoblotted using the indicated antibodies.

**Table S1.** Mutations and gene amplification found by OncoPanel sequencing in 11 clinically diagnosed never/light smokers with SCLC.
