# Supplementary material for: Genomic and pathological heterogeneity in clinically diagnosed small cell lung cancer in never/light smokers identifies therapeutically targetable alterations
Source: Mol Oncol. 2020 Nov 25;15(1):27–42. doi: 10.1002/1878-0261.12673 (PMC7782083; doi:10.1002/1878-0261.12673)
Supplement: Supplementary file 1 — Fig. S1. Treatment timeline: Dynamic contrast‐enhanced CT scan and FDG‐PET image are shown. [file MOL2-15-27-s001.pptx]

## Slide 1
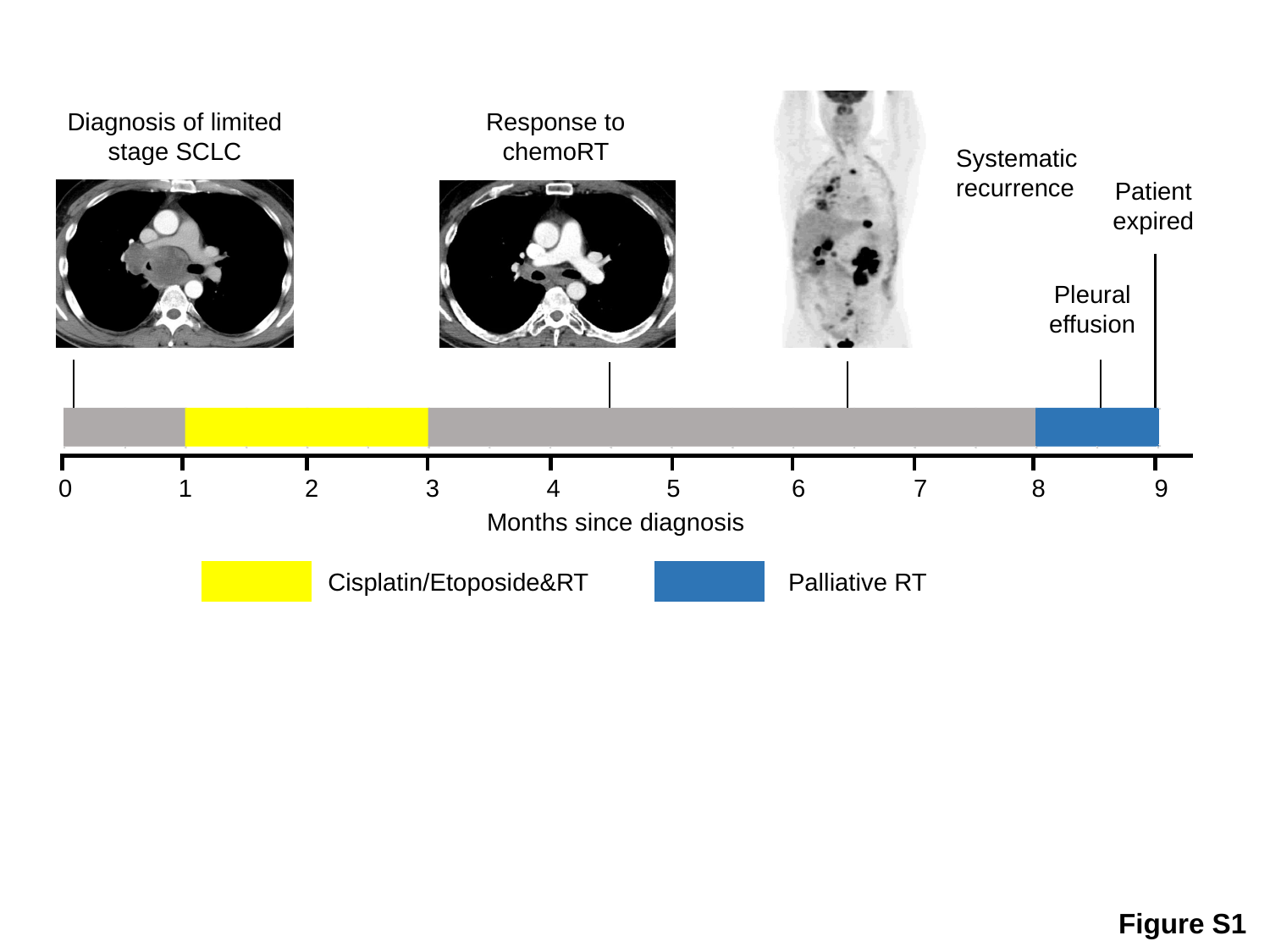

Diagnosis of limited stage SCLC
Response to chemoRT
Systematic recurrence
Patient expired
Pleural effusion
0
1
2
3
4
5
6
7
8
9
Months since diagnosis
Cisplatin/Etoposide&RT
Palliative RT
| |
| --- |
| |
| --- |
Figure S1
