# Supplementary material for: Genomic and pathological heterogeneity in clinically diagnosed small cell lung cancer in never/light smokers identifies therapeutically targetable alterations
Source: Mol Oncol. 2020 Nov 25;15(1):27–42. doi: 10.1002/1878-0261.12673 (PMC7782083; doi:10.1002/1878-0261.12673)
Supplement: Supplementary file 2 — Fig. S2. Characterization of NRAS mutant lung cancer cell lines. (A) Flow cytometric analysis of NCAM/EpCAM expression on DFCI168. (B) (left) The lysates from nuclear fractions of lung cancer cell lines were examined for RB, pRB (Ser807/811) and lamin B (loading control). (right) IHC image of DFCI168 PDX stained with RB. Scale bar 50 µm. (C) The activation of PI3K/Akt/mTOR and MEK/ERK pathways in the NRAS mutant lung cancer cell lines was assessed by western blot. (D) Immunofluorescence images of DFCI168 stained with phalloidin (green) and VIM (red). Scale bars 100 µm. [file MOL2-15-27-s002.pptx]

## Slide 1
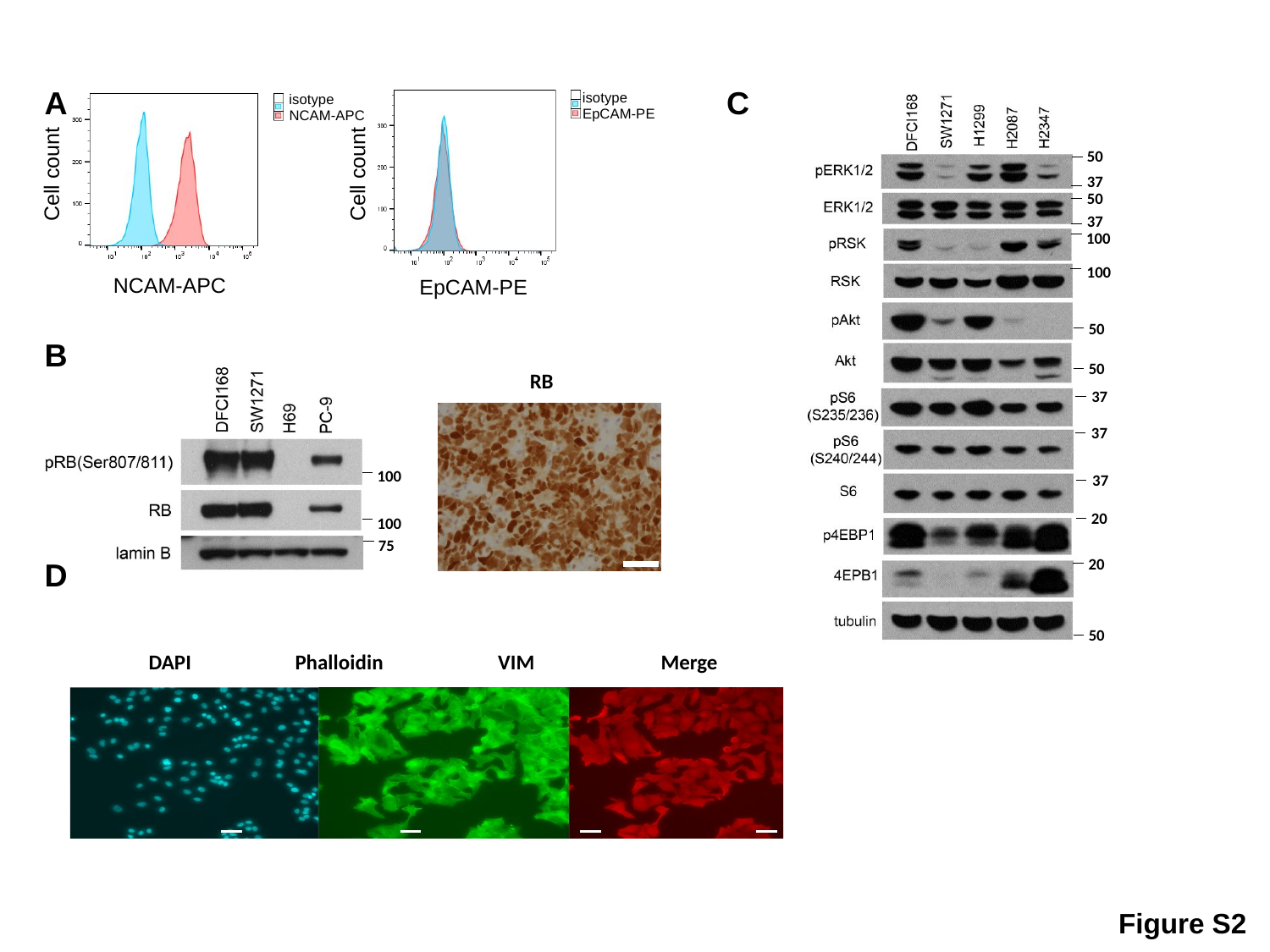

isotype
EpCAM-PE
EpCAM-PE
isotype
NCAM-APC
NCAM-APC
A
C
50
Cell count
Cell count
37
50
37
100
100
50
B
100
100
75
50
RB
37
37
37
20
20
D
50
VIM
DAPI
Phalloidin
Merge
Figure S2
