# Supplementary figures and images for: Genomic and pathological heterogeneity in clinically diagnosed small cell lung cancer in never/light smokers identifies therapeutically targetable alterations
Source: Mol Oncol. 2020 Nov 25;15(1):27–42. doi: 10.1002/1878-0261.12673 (PMC7782083; doi:10.1002/1878-0261.12673)

## Slide 1
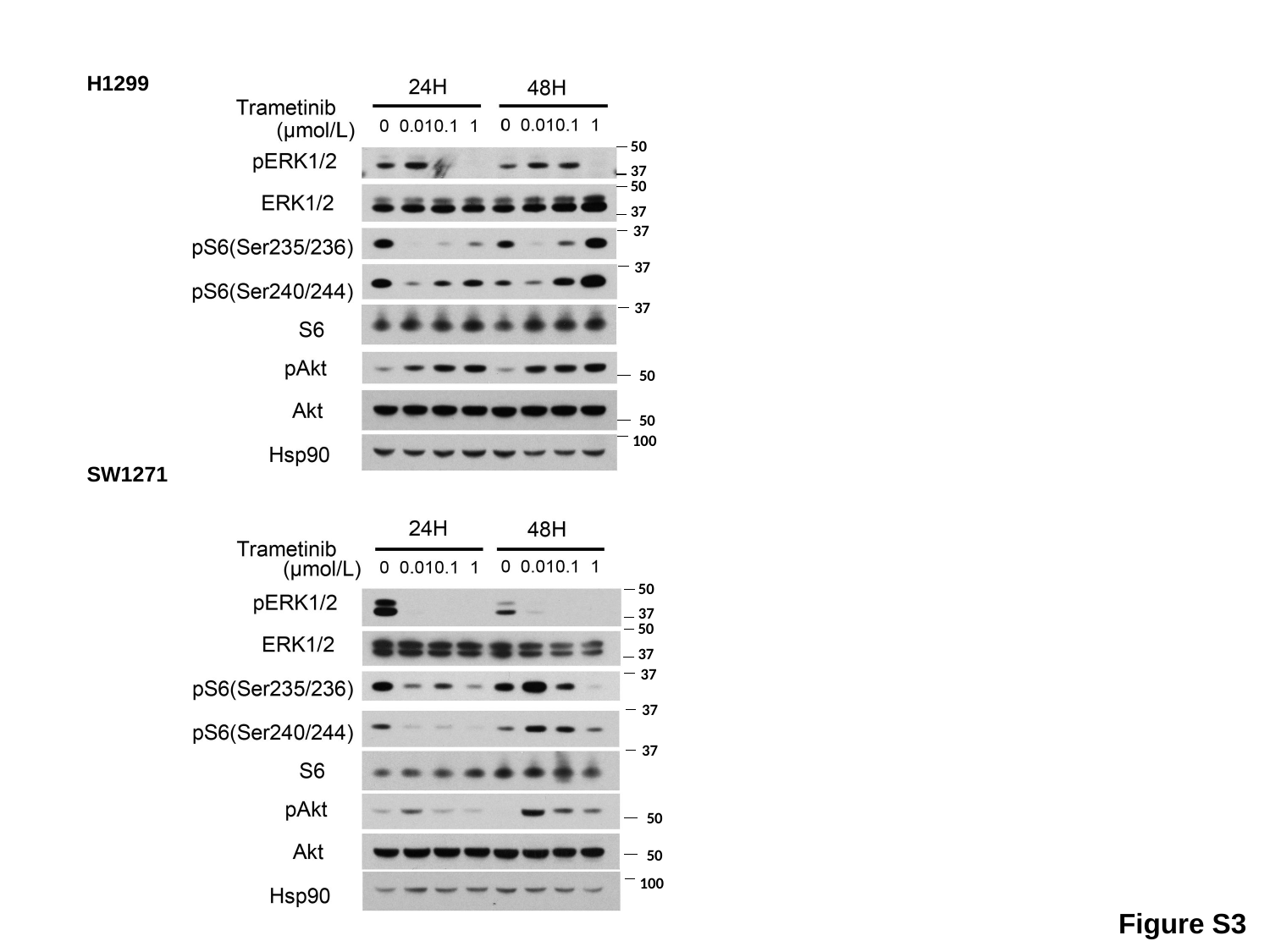

H1299
50
37
50
37
37
37
37
50
50
100
SW1271
50
37
50
37
37
37
37
50
50
100
Figure S3

Supplement: Supplementary file 3 — Fig. S3. H1299 and SW1271 were treated with trametinib at the indicated concentration for the indicated times. The cell extracts were immunoblotted using the indicated antibodies. [file MOL2-15-27-s003.pptx]

## Slide 1
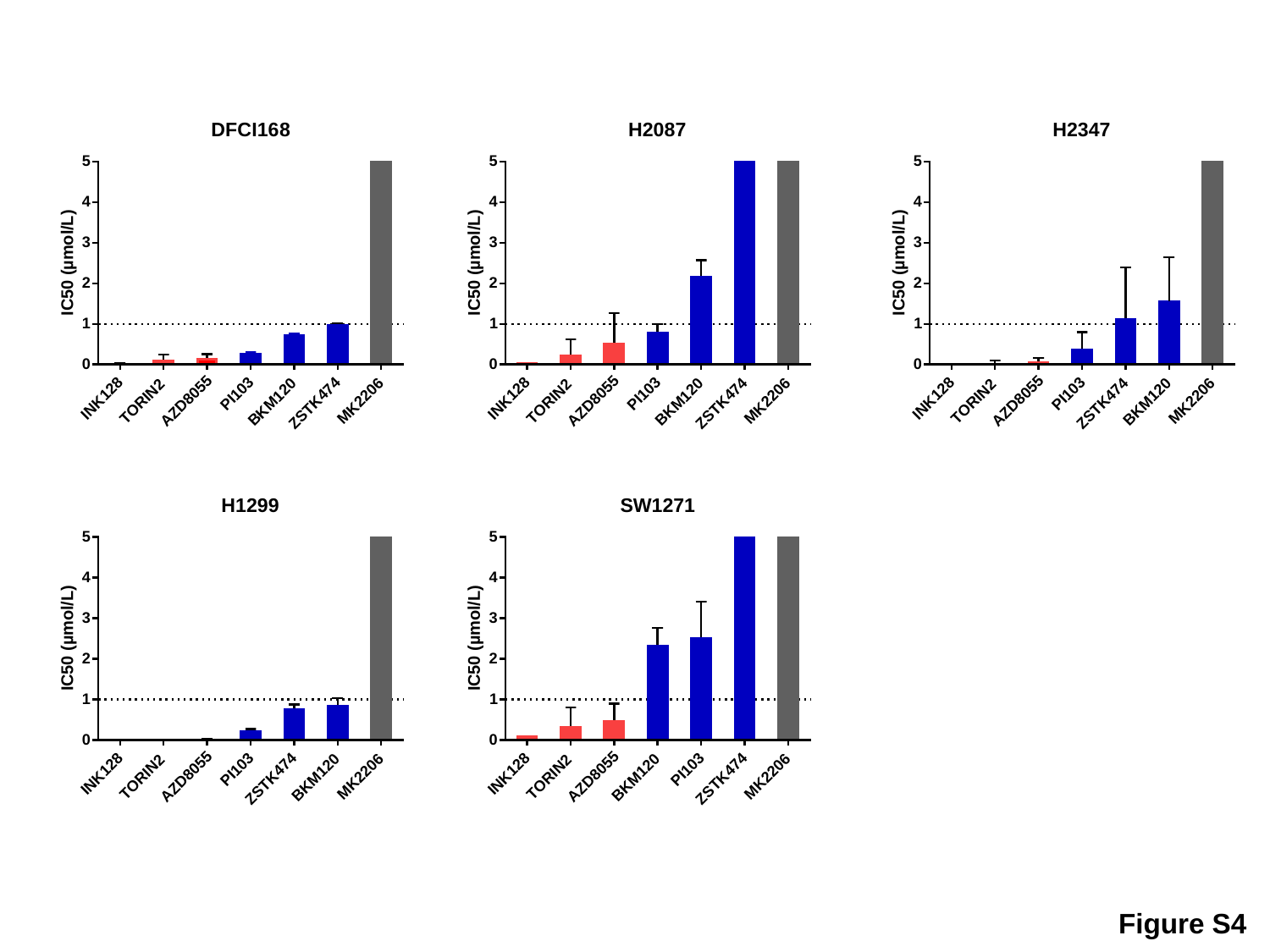

Figure S4

Supplement: Supplementary file 4 — Fig. S4. The comparison of IC50 values of various inhibitors for NRAS mutant lung cancer cell lines after 72 h of treatment. The results were obtained from three independent experiments, and the bar represents the mean ± SD. [file MOL2-15-27-s004.pptx]

## Slide 1
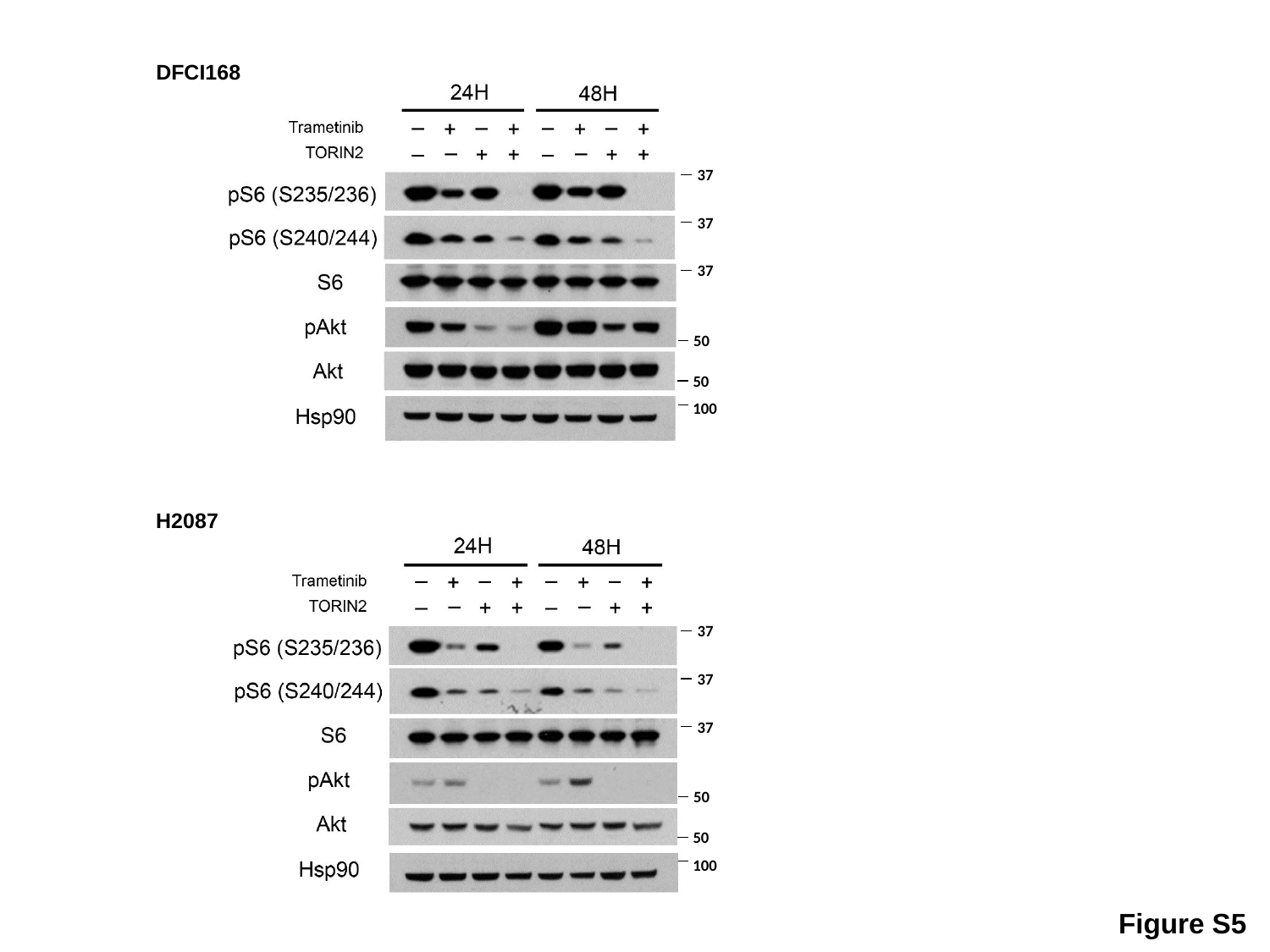

DFCI168
37
37
37
50
50
100
H2087
37
37
37
50
50
100
Figure S5

Supplement: Supplementary file 5 — Fig. S5. DFCI168 and H2087 were treated with 100 nm of trametinib/torin2 either alone or in combination for the indicated times. The cell extracts were immunoblotted using the indicated antibodies. [file MOL2-15-27-s005.pptx]
