# Supplementary material for: Genomic and pathological heterogeneity in clinically diagnosed small cell lung cancer in never/light smokers identifies therapeutically targetable alterations
Source: Mol Oncol. 2020 Nov 25;15(1):27–42. doi: 10.1002/1878-0261.12673 (PMC7782083; doi:10.1002/1878-0261.12673)
Supplement: Supplementary file 6 — Table S1. Mutations and gene amplification found by OncoPanel sequencing in 11 clinically diagnosed never/light smokers with SCLC. [file MOL2-15-27-s006.pptx]

## Slide 1
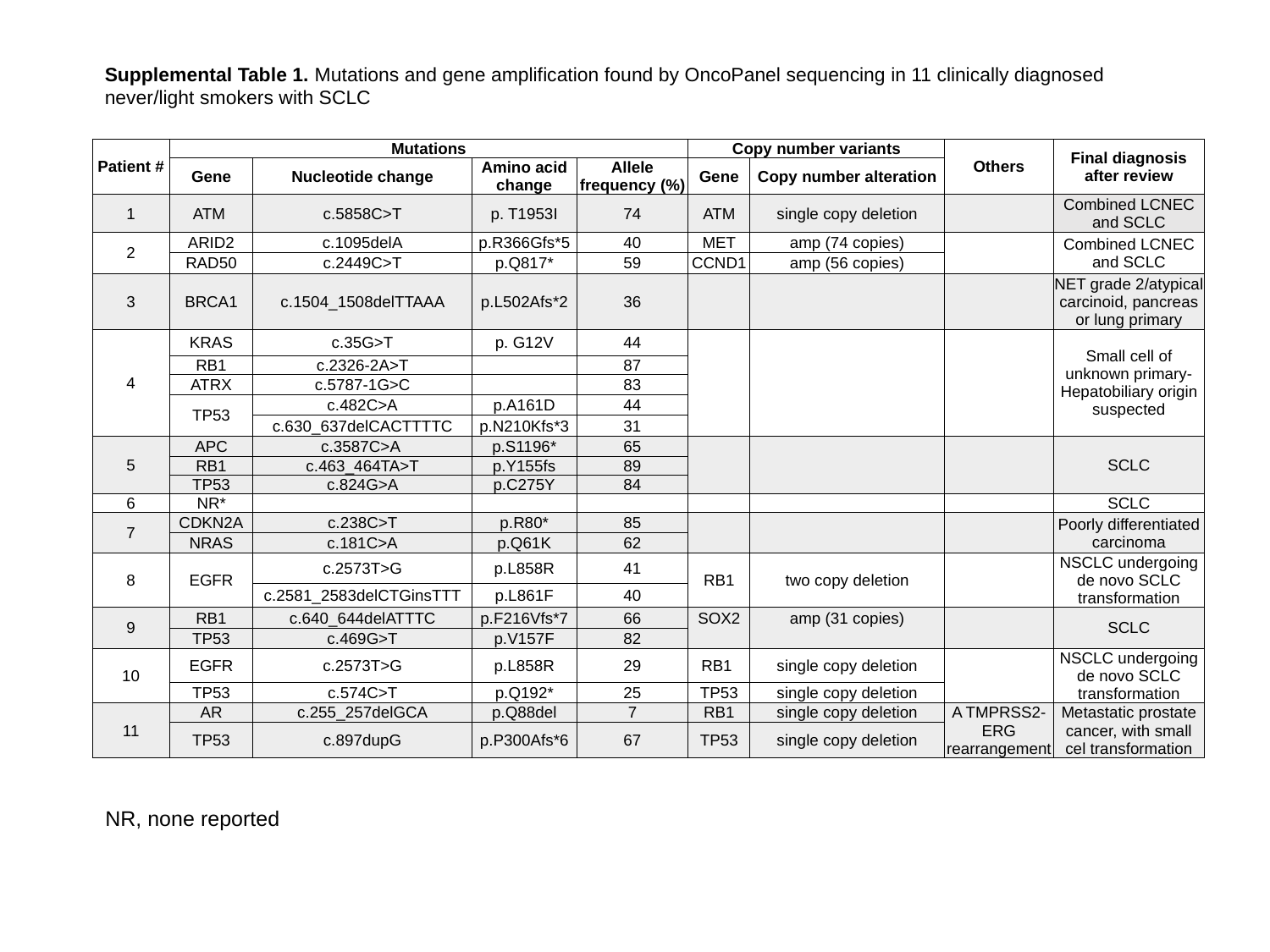

Supplemental Table 1. Mutations and gene amplification found by OncoPanel sequencing in 11 clinically diagnosed never/light smokers with SCLC
| Patient # | Mutations | | | | Copy number variants | | Others | Final diagnosis after review |
| --- | --- | --- | --- | --- | --- | --- | --- | --- |
| | Gene | Nucleotide change | Amino acid change | Allele frequency (%) | Gene | Copy number alteration | | |
| 1 | ATM | c.5858C>T | p. T1953I | 74 | ATM | single copy deletion | | Combined LCNEC and SCLC |
| 2 | ARID2 | c.1095delA | p.R366Gfs\*5 | 40 | MET | amp (74 copies) | | Combined LCNEC and SCLC |
| | RAD50 | c.2449C>T | p.Q817\* | 59 | CCND1 | amp (56 copies) | | |
| 3 | BRCA1 | c.1504\_1508delTTAAA | p.L502Afs\*2 | 36 | | | | NET grade 2/atypical carcinoid, pancreas or lung primary |
| 4 | KRAS | c.35G>T | p. G12V | 44 | | | | Small cell of unknown primary- Hepatobiliary origin suspected |
| | RB1 | c.2326-2A>T | | 87 | | | | |
| | ATRX | c.5787-1G>C | | 83 | | | | |
| | TP53 | c.482C>A | p.A161D | 44 | | | | |
| | | c.630\_637delCACTTTTC | p.N210Kfs\*3 | 31 | | | | |
| 5 | APC | c.3587C>A | p.S1196\* | 65 | | | | SCLC |
| | RB1 | c.463\_464TA>T | p.Y155fs | 89 | | | | |
| | TP53 | c.824G>A | p.C275Y | 84 | | | | |
| 6 | NR\* | | | | | | | SCLC |
| 7 | CDKN2A | c.238C>T | p.R80\* | 85 | | | | Poorly differentiated carcinoma |
| | NRAS | c.181C>A | p.Q61K | 62 | | | | |
| 8 | EGFR | c.2573T>G | p.L858R | 41 | RB1 | two copy deletion | | NSCLC undergoing de novo SCLC transformation |
| | | c.2581\_2583delCTGinsTTT | p.L861F | 40 | | | | |
| 9 | RB1 | c.640\_644delATTTC | p.F216Vfs\*7 | 66 | SOX2 | amp (31 copies) | | SCLC |
| | TP53 | c.469G>T | p.V157F | 82 | | | | |
| 10 | EGFR | c.2573T>G | p.L858R | 29 | RB1 | single copy deletion | | NSCLC undergoing de novo SCLC transformation |
| | TP53 | c.574C>T | p.Q192\* | 25 | TP53 | single copy deletion | | |
| 11 | AR | c.255\_257delGCA | p.Q88del | 7 | RB1 | single copy deletion | A TMPRSS2-ERG rearrangement | Metastatic prostate cancer, with small cel transformation |
| | TP53 | c.897dupG | p.P300Afs\*6 | 67 | TP53 | single copy deletion | | |
NR, none reported
